# Progesterone Attenuates Brain Inflammatory Response and Inflammation-Induced Increase in Immature Myeloid Cells in a Mouse Model

**DOI:** 10.1007/s10753-020-01390-y

**Published:** 2021-01-06

**Authors:** Ola Gutzeit, Linoy Segal, Ben Korin, Roee Iluz, Nizar Khatib, Fadwa Dabbah-Assadi, Yuval Ginsberg, Ofer Fainaru, Michael G. Ross, Zeev Weiner, Ron Beloosesky

**Affiliations:** 1 Department of Obstetrics and Gynecology, Rambam Health Care Campus, Rambam Medical Center, 8 Ha’alya St., 3109601 Haifa, Israel; 2 Department of Neuroscience, Rappaport Faculty of Medicine, Technion - Israel Institute of Technology, Haifa, Israel; 3 Department of Immunology, Rappaport Faculty of Medicine, Technion - Israel Institute of Technology, Haifa, Israel; 4 Department of Obstetrics and Gynecology, Harbor-UCLA Medical Center, Torrance, CA USA

**Keywords:** brain immune system, immature myeloid cells, inflammation, microglia, progesterone

## Abstract

Progesterone has been shown to regulate immunity during pregnancy, and progesterone administration may reduce inflammation-induced preterm labor. We sought to determine the maternal brain immune response to LPS-induced inflammation in pregnant and non-pregnant mice and whether additional progesterone supplementation attenuates this response. Pregnant (P: *n* = 9) and non-pregnant mice (NP: *n* = 9) were randomized to pretreatment with vaginal progesterone/carrier (Replens), daily from days 13 to 16. On days 15 and 16, LPS/saline was administered by intraperitoneal injection (Replens + saline *n* = 3; Replens + LPS *n* = 3; progesterone + LPS *n* = 3). Mice were sacrificed on day 16 and maternal serum analyzed for IL-6 levels and brains analyzed for nNOS, NF-kB, IL-6 protein levels and for immature myeloid cells (IMCs) and microglial activity. LPS significantly increased brain nNOS, NF-kB, and IL-6 in both NP and P mice, with significantly greater responses in P mice. In both NP and P groups, progesterone significantly attenuated LPS-induced increase of nNOS and NF-kB, however with no effect on serum IL-6. In the NP brains, LPS significantly increased IMC population and progesterone reduced the IMC phenotype to levels similar to controls. In P mice, neither LPS nor LPS + progesterone altered the brain IMC population. LPS significantly increased the microglial activity in both NP and P groups, which was attenuated by progesterone. Progesterone attenuates brain inflammatory response to LPS in both NP and P mice although it has no effect on systemic inflammation. In NP mice, progesterone attenuated the increase in brain IMC following LPS administration. Our results suggest that endogenous progesterone during pregnancy may protect the brain from LPS-induced inflammation.

## INTRODUCTION

Pregnancy represents a unique immune tolerance [1]. Maintenance of pregnancy represents a challenge for the maternal immune system since it has to defend against pathogens while tolerating paternal alloantigens expressed in fetal and placental tissues [2]. Immune imbalance during pregnancy may contribute to pathological conditions such as preeclampsia, recurrent spontaneous abortion, and intrauterine growth restriction [3]. Consistent with immune suppression, during pregnancy, a portion of women with cell-mediated autoimmune diseases (*e.g.*, multiple sclerosis) evidence reduced symptoms, especially during the third trimester [4].

Progesterone is a pivotal hormone in pregnancy, as it maintains uterine quiescence [5]. Progesterone supplementation is recommended by the American College of Obstetrics and Gynecology for prevention of preterm birth in select populations [6]. There are two types of progesterone currently used for prevention of preterm birth: (1) weekly intramuscular injections of 17α-OHPC and (2) daily administration of natural micronized progesterone. 17α-OHPC has been shown to be effective in preventing PTB in pregnant women with a history of PTB. Micronized progesterone has been shown to be effective for women with short cervical length [7]. Natural progesterone was chosen for this study because natural progesterone but not 17α-OHPC has been shown to have anti-inflammatory effects at the murine maternal-fetal interface [8]. As inflammation represents a putative mechanism for preterm labor, anti-inflammatory properties may be intrinsic to progesterone prevention of preterm birth. Several studies have shown that progesterone may repress NF-κβ and pro-inflammatory cytokine synthesis, such as TNF-α [9].

In adults, progesterone reduces neuroinflammation, oxidative stress, and brain damage following traumatic brain injury [10]. Among mothers who received vaginal progesterone during pregnancy, the OPPTIMUM study [11] reported a reduction in neonatal brain injuries on cerebral ultrasound scanning. Little is known regarding the maternal brain immune system during pregnancy, and its ability to respond to inflammation under this unique hormonal environment. In this study, we investigated the effect of pregnancy and progesterone treatment on mouse brain immune responses to systemic LPS-induced inflammation.

## EXPERIMENTAL PROCEDURES

### Experimental Models

Animal studies were carried out using 6–8-week-old pregnant and NP ICR (CD-1) female mice (Harlan Laboratories, Jerusalem, Israel). Pregnant mice were supplied on day 8 of pregnancy and allowed to acclimate for 5 days prior to initiating experiments. Pregnant (P, *n* = 9) mice were randomly pretreated with vaginal progesterone (1 mg/day) (Sigma, St. Louis, MO, USA) or carrier (Replens) from day 13 to day 16 of gestation. Lipopolysaccharide (LPS) 30 μg in 0.1 mL (*Escherichia coli* serotype 0111; B4, Calbiochem; Merck, Darmstadt, Germany) or saline (0.1 mL) were administered intraperitoneally on days 15 and 16 (12 h apart) (P: Replens + saline *n* = 3; Replens + LPS *n* = 3; progesterone + LPS *n* = 3). Non-pregnant mice (NP; *n* = 9) were identically randomized and treated (PN: Replens + saline *n* = 3; Replens + LPS *n* = 3; progesterone + LPS *n* = 3). Control animals are defined as P or NP receiving Replens + saline. Four hours following the last LPS/saline injection, mice were sacrificed by CO_2_ inhalation, blood was collected from the heart, and brains were harvested as described below.

### Brain Single-Cell Dissociation

Mice were perfused through the left ventricle with ice-cold PBS without magnesium and calcium. Brains were extracted and immune cells were isolated as described previously [12], by centrifugation through a 70–30% Percoll (Sigma-Aldrich, GE Healthcare Bio-Sciences). Following extraction, brains were transferred to RPMI-1640 (Sigma-Aldrich) and dissociated by a dounce homogenizer. The cell suspension, with 30% Percoll, was layered on top of the 70% Percoll solution in PBS. Following centrifugation (30 min, 500 *g*, 18 °C, minimal brake), the cells at a 70–30% interphase were taken and washed with PBS. The cell pellet was suspended in 1 mL staining buffer (1% bovine serum albumin and 0.05% sodium azide in PBS) and washed one more time.

### Flow Cytometry

Immunostaining was performed in the presence of rat anti-mouse Fc receptor III/II (FcgammaRIII/II) (CD16/32; Pharmingen, San Diego, CA, USA), by incubating the cells with monoclonal antibodies for 30 min on ice. Staining reagents included fluorochrome (PE, PerCP Cy5.5 or PE Cy7) labeled anti-CD11b, CD45, Gr1 (eBioscience, San Diego, CA, USA). Flow cytometry analysis was done using the FlowJo 10.1r5 software (Tree Star). Double discrimination of cells was performed prior to every analysis.

### Protein Extraction and Western Blotting

The maternal brains were homogenized in lysis buffer containing 2% SDS, 10% glycerol, 2% 2-mercaptoethanol, and 0.002% bromophenol blue in 75 mm Tris–HCl. The samples were heated at 95 °C for 10 min before separating on 10% Tris/glycine/SDS acrylamide gels. The proteins were subsequently trans-blotted to polyvinylidene difluoride membranes and blocked in 5% dry milk for 2 h at room temperature. The membrane was incubated with rat anti-nNOS, NF-kB, and IL-6 (Santa Cruz Company, USA) for 2 h at 37 °C. After three washes with TBS/0.05% Tween-20, the membrane was incubated with a horseradish peroxidase–conjugated goat anti-rat antibody (Santa Cruz) for 1 h at 37 °C. Protein signal was visualized using the Super Signal West Pico Chemiluminescent Substrate (PIERCE Company, Waltham, MA, USA) and detected with Imaging System (Syngene Company, Frederick, MD, USA). β-Actin protein was visualized and detected as above. The ratio between nNOS, NF-kB, and IL-6 actin density for each sample was determined using a densitometer. Commercial enzyme-linked immunosorbent assay (R&D Systems, Minneapolis, MN) kits were used to determine blood protein levels of the cytokines IL-6 (R6000) according to manufacturer’s protocol and as previously described [13]. The minimum detectable level was <10 pg/mL with both intra-assay and inter-assay variations <10%.

### Statistical Analysis

All results are expressed as means ± SD using one-way analysis of variance followed by *post hoc* tests for pairwise comparisons (Holm-Sidak method). Differences were considered to be significant at *p* < 0.05.

### Ethics Statement

This study was carried out in strict accordance with the recommendations in the Guide for the Care and Use of Laboratory Animals of the National Institutes of Health. All animal procedures were performed in compliance with the inspection committee on the constitution of the animal experimentation at the Technion (IL-117-08-2017).

## RESULTS

### Maternal Systemic Inflammation: Serum IL-6

Basal levels of IL-6 were similar in NP and P mice. LPS significantly increased IL-6 serum levels in NP and P mice (NP: 10.3 ± 0.3 *vs.* 2947 ± 62 pg/mL, *p* < 0.01; P: 11.4 ± 3.1 *vs.* 2501 ± 630 pg/mL, *p* < 0.01). Progesterone pretreatment prior to LPS injection had no significant effect on IL-6 blood levels in NP or P groups.

### Basal Brain Inflammatory Pathways and Cytokines

**Fig. 1:**
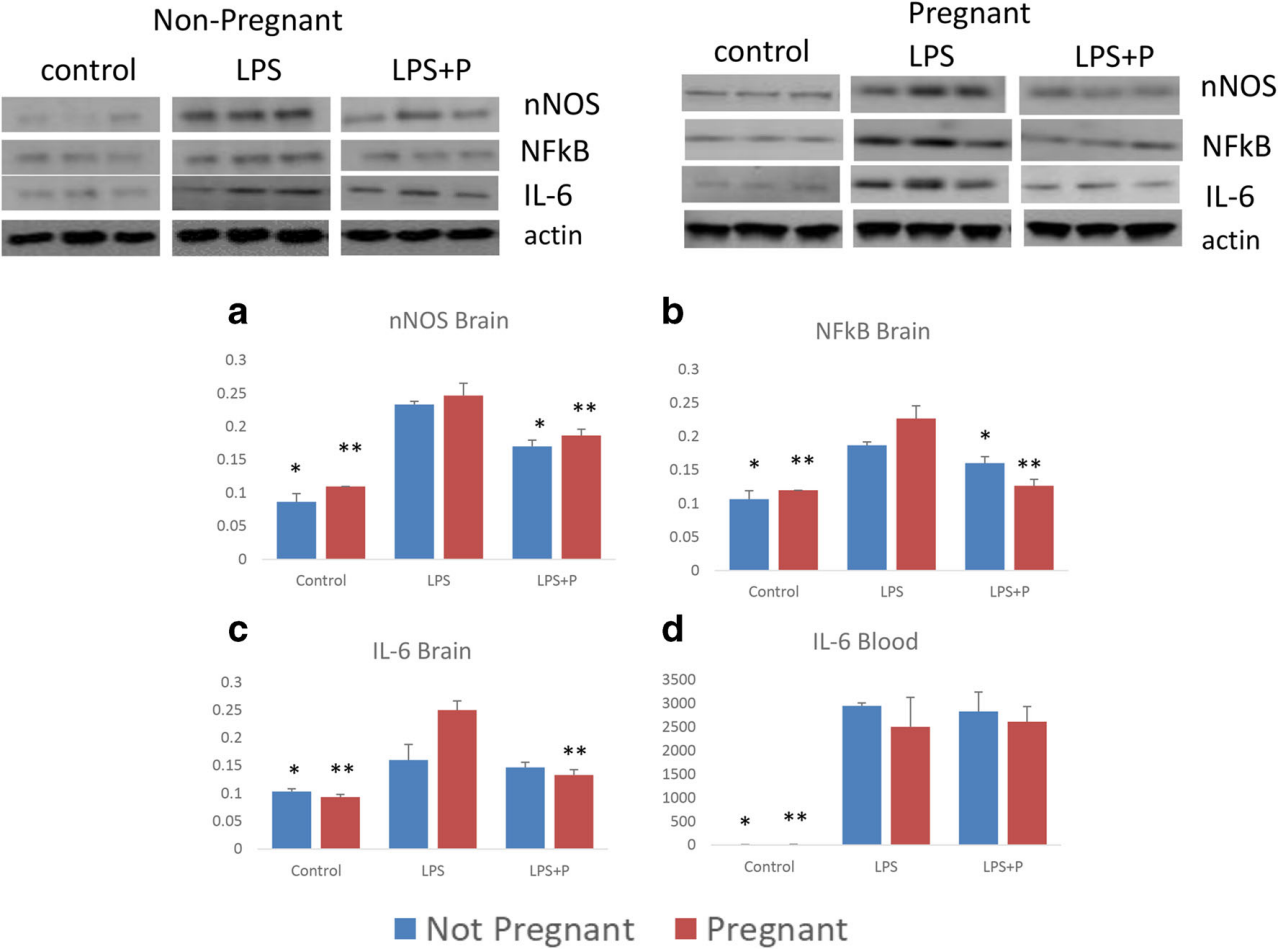
Non-pregnant and pregnant mouse brain inflammatory pathways and brain and blood cytokine at basal state, following inflammation with or without progesterone pretreatment. Brain mean protein levels (u) of **a** nNOS, **b** NF-kB, **c** IL-6. **d** blood IL-6 protein levels. *p < 0.05, compared to the non-pregnant LPS group; **p < 0.05, compared to the pregnant LPS group.

The brain protein levels of nNOS, NF-kB, and IL-6 were similar between NP and P control mice (Table 1 and Fig. 1a–d). LPS significantly increased brain nNOS, NF-kB, and IL-6 protein levels in both NP and P ICR mice compared to control (Table 1 and Fig. 1a–d). Following LPS, P mice had significantly more robust inflammatory response compared to NP mice in levels of NF-kB and IL-6 (Table 1), though there was no difference in the nNOS response.

**Table 1:** Non-Pregnant and Pregnant Mice Brain nNOS, NF-kB, And IL-6 At Basal State, Following Inflammation With Or Without Progesterone Pretreatment

Brain cytokine	Group		Mean (u)	Standard deviation	*p* value compared to LPS	*p* value compared to non-pregnant
nNOS	Non-pregnant	Control	0.09	0.01	0.01 >	
		LPS	0.23	0.01		
		LPS + P	0.17	0.01	0.01 >	
	Pregnant	Control	0.11	0.01	0.01 >	0.06
		LPS	0.24	0.01		0.38
		LPS + P	0.18	0.01	0.01 >	0.13
NF-kB	Non-pregnant	Control	0.11	0.01	0.01 >	
		LPS	0.19	0.01		
		LPS + P	0.16	0.01	0.01 >	
	Pregnant	Control	0.12	0.01	0.01 >	0.05 >
		LPS	0.22	0.02		0.05 >
		LPS + P	0.12	0.01	0.01 >	0.01 >
IL-6	Non-pregnant	Control	0.1	0.01	0.05 >	
		LPS	0.16	0.03		
		LPS + P	0.14	0.01	0.56	
	Pregnant	Control	0.09	0.01	0.01 >	0.1
		LPS	0.25	0.02		0.05 >
		LPS + P	0.13	0.01	0.01 >	0.23

*LPS*, lipopolysaccharide, *P*, progesterone

Progesterone pretreatment significantly reduced the increase in nNOS and NF-kB brain levels in LPS treatment groups in both NP and P mice compared to LPS-treated controls (Table 1). Progesterone reduced the LPS increase in brain IL-6 levels in P mice but not in NP mice (Table 1).

### Brain IMCs

**Fig. 2:**
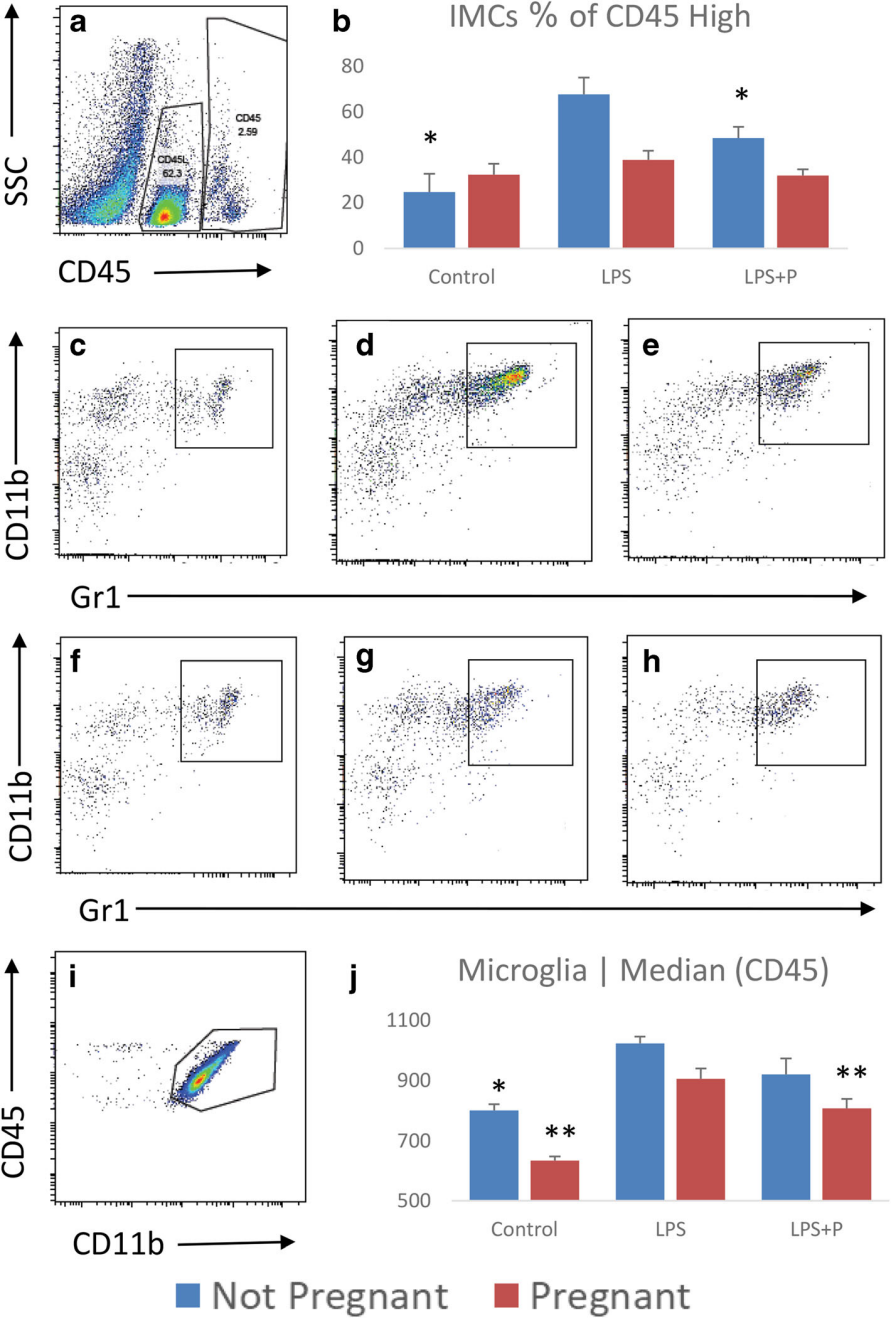
Non-pregnant and pregnant mouse brain IMCs and microglial activity at basal state, following inflammation with or without progesterone pretreatment. a microglia was gated as CD45^low^; brain hematopoietic cells were gated as CD45^high^. **b–d** CD45^high^CD11b^+^Gr1^+^ IMCs in non-pregnant mice: Control, LPS, LPS + P respectively. e–**g** CD45^high^CD11b^+^Gr1^+^ IMCs in pregnant mice: Control, LPS, LPS + P respectively. h the percentage of CD11b^+^Gr1^+^ IMCs out of CD45^high^ hematopoietic cells are plotted as mean ± SD. i microglial activity measured as medial CD45 intensity in CD45^low^ cells plotted as mean ± SD. *p < 0.05, compared to the non-pregnant LPS group; **p < 0.05, compared to the pregnant LPS group.

To evaluate the brain immune response, single cells isolated from the brain were immunostained and analyzed by flow cytometry. We analyzed the CD45^high^ infiltrating hematopoietic brain population for the percentage of immature myeloid cells (IMCs) (CD45^high^, CD11b^+^, Gr1^+^; Fig. 2a–h). There was no significant difference in brain IMC population in NP and P mice. In NP mice, LPS treatment significantly increased brain IMC population compared to untreated controls, while progesterone pretreatment in LPS-treated mice reduced this increase (Table 2). In the P group, neither LPS nor LPS and progesterone altered brain IMC population.

**Table 2:** Non-Pregnant And Pregnant Mice Brain IMCs And Microglial Activity At Basal State, Following Inflammation With Or Without Progesterone Pretreatment

Brain immune system cells	Group		Mean (%)	Standard deviation	*p* value compered to LPS	*p* value compered to non-pregnant
IMCs	Non-pregnant	Control	24.7	8.07	0.01 >	
		LPS	67.8	7.45		
		LPS + P	48.6	4.94	0.05 >	
	Pregnant	Control	32.46	4.78	0.07	0.19
		LPS	38.86	4.07		0.01 >
		LPS + P	31.96	2.79	0.06	0.05 >
Microglia Median (CD45)	Non-pregnant	Control	801.33	20.8	0.01 >	
		LPS	1023.66	22.95		
		LPS + P	920	53.23	0.06	
	Pregnant	Control	634.33	13.67	0.01 >	0.01 >
		LPS	905.66	34.39		0.01 >
		LPS + P	808	31.02	0.05 >	0.06

*IMC*, immature myeloid cells; *LPS*, lipopolysaccharide; *P*, progesterone

### Microglial Activity

Microglia were defined as CD45^low^CD11b^+^ cells and microglial activity was measured according to the median medial CD45 intensity (Fig. 2i–j). Basal microglial activity in the NP group was significantly higher than in the N group (Table 2). LPS treatment significantly increased the microglial activity in both the NP and P mice as indicated by the intensity of CD45 expression (Table 2). Progesterone pretreatment significantly decreased microglial activity compared to controls in P mice (Table 2). In NP mice, there was a trend for decreased microglial activity, but it did not reach statistical significance.

## DISCUSSION

Previous studies have demonstrated that LPS induces maternal systemic inflammation and impairs fetal and offspring brain development [13–16]. Few studies have investigated the effect of inflammation on the maternal brain and compared P and NP responses [17, 18]. In the present study, we compared for the first time the effect of LPS-induced systemic inflammation and natural progesterone supplementation on the brain of P and NP mice. We demonstrated that progesterone attenuates brain inflammatory response following LPS in both NP and P mice although it has no effect on systemic (IL-6) inflammation. In NP mice, progesterone attenuated the increase in brain IMC following LPS administration.

During pregnancy there is an increase in endogenous progesterone, with peak levels in the third trimester [19]. Progesterone has an immune suppressive effect on the innate immune response [20, 21]. Progesterone receptors have been identified in macrophages, dendritic cells, and lymphocytes [22]. The brain innate immune system consists of resident microglia and infiltrating and resident myeloid and lymphoid cells. It was previously demonstrated that microglia express progesterone receptor [23]. Nestorone, a synthetic progestin with high affinity to progesterone receptor, provides neuroprotection and enhances myelin repair in chronic demyelinating lesions induced by feeding cuprizone to female mice [24]. The remyelination effect is progesterone receptor dependent, as homozygous progesterone receptor knockout mice, unlike wild-type mice, do not experience remyelination and heterozygous progesterone receptor knockout mice experience curtailed remyelination upon progesterone [24].

Our results show that in P mice, with high endogenous progesterone, the basal activity of resident microglia cells is significantly lower than the NP mice, with no significant difference in basal IMC population or inflammation mediators compared to NP mice. These results may imply a selective influence of progesterone in maternal brain through progesterone receptors in the resident immune cells. On other hand, there is no effect of progesterone on cells and inflammation mediators that originate in the periphery and cross the blood–brain barrier (BBB).

LPS is a potent activator of innate immunity by its activation of toll-like receptor 4 expressed on innate immune cells including microglia. Systemic administration of LPS induces robust neuroinflammation and microglial activation despite the poor brain penetration [25]. Cytokines are primarily synthesized in the periphery, cross the BBB, and induce microglia to produce immune mediators [25]. Additionally, LPS increases BBB permeability [25]. We demonstrated that progesterone pretreatment attenuated the increase in brain pro-inflammatory mediators following systemic LPS-induced inflammation, with no effect on the increase in blood IL-6. This may be explained by the predominant effect of progesterone on production of immune mediators by the brain-resident innate immune system in a response to the cytokine infiltration from the periphery. Our results support a previous study in the wobbler mouse model of motor neuron degeneration, demonstrating that Nestorone downregulates NF-kB, TLR4, and nNOS proinflammatory factors as well as microglial CD11b expression at the mRNA level [26].

IMCs are bone marrow–derived cells that normally differentiate into granulocytes, macrophages, and dendritic cells (DCs), but expand in pathological conditions such as malignancy [27]. We recently demonstrated that progesterone supplementation attenuated the increase in placental IMCs following LPS-induced inflammation [28]. It was previously demonstrated that those unique cells are also present in the naïve mouse brain [29]. Here we demonstrated for the first time that the normal prevalence of brain-resident IMCs in NP and P mice is similar. Systemic inflammation, however, significantly increased brain IMC population in NP mice but not in P mice. This effect suggests that endogenous progesterone which is abundant in pregnancy may protect the maternal brain from infiltration of IMCs after systemic inflammation. In NP mice, progesterone supplementation attenuated the increase in these brain immune cells. Our findings suggest that progesterone predominantly affects the brain-resident innate immune response in a response to systemic inflammation.

Animal model of systemic inflammation effect on NP and P mice enables us to investigate the molecular maternal brain immune response. Although vaginal natural progesterone supplementation does not affect maternal peripheral immune response, it has significant effect on the maternal CNS immune system. Those changes may be effected by dose, route of administration, and length of exposure. What is the long-term effect of those changes and whether those changes have effect on brain function? There are two types of progesterone currently used for prevention of preterm birth: 17α-OHPC (synthetic) and natural micronized progesterone. There are chemical, biological, and pharmacologic differences between the two types of progesterone; what is the effect of 17α-OHPC on maternal brain immune response is an interesting question for future studies.

The strength of our study is in the novel finding of a progesterone attenuation effect on maternal brain immune response following systemic inflammation. The limitation of this study is that all data were obtained from animal models and modest sample size.

We demonstrated that progesterone supplementation attenuates brain inflammatory response to LPS in both NP and P mice although it has no effect on systemic (IL-6) inflammation. In NP mice, progesterone attenuates the increase in brain IMC following LPS administration. Our results suggest that endogenous progesterone during pregnancy may protect the brain from LPS-induced inflammation.

## Data Availability

All data and materials as well as software application support our published claims and comply with field standards.
